# Publisher Correction: IL-17 signalling is critical for controlling subcutaneous adipose tissue dynamics and parasite burden during chronic murine *Trypanosoma brucei* infection

**DOI:** 10.1038/s41467-024-46299-4

**Published:** 2024-02-28

**Authors:** Matthew C. Sinton, Praveena R. G. Chandrasegaran, Paul Capewell, Anneli Cooper, Alex Girard, John Ogunsola, Georgia Perona-Wright, Dieudonné M Ngoyi, Nono Kuispond, Bruno Bucheton, Mamadou Camara, Shingo Kajimura, Cécile Bénézech, Neil A. Mabbott, Annette MacLeod, Juan F. Quintana

**Affiliations:** 1 Wellcome Centre for Integrative Parasitology, University of Glasgow, Glasgow, UK; 2 School of Biodiversity, One Health and Veterinary Medicine, University of Glasgow, Glasgow, UK; 3 Division of Cardiovascular Science, University of Manchester, Manchester, UK; 4 School of Infection and Immunity, University of Glasgow, Glasgow, UK; 5 Department of Parasitology, National Institute of Biomedical Research, Kinshasa, Democratic Republic of Congo; 6 Member of TrypanoGEN, Kinshasa, Democratic Republic of Congo; 7 Institut de Recherche pour le Développement, Unité Mixte de Recherche IRD-CIRAD 177, Campus International de Baillarguet, Montpellier, France; 8 Programme National de Lutte contre la Trypanosomiase Humaine Africaine, Ministère de la Santé, Conakry, Guinea; 9 Division of Endocrinology, Diabetes and Metabolism, Beth Israel Deaconess Medical Center and Harvard Medical School, Boston, MA USA; 10 Howard Hughes Medical Institute, Chevy Chase, MD USA; 11 Centre for Cardiovascular Science, University of Edinburgh, Edinburgh, EH16 4TJ Scotland UK; 12 The Roslin Institute and Royal (Dick) School of Veterinary Studies, University of Edinburgh, Edinburgh, UK; 13 Lydia Becker Institute of Immunology and Inflammation, University of Manchester, Manchester, UK; 14 Division of Immunology, Immunity to Infection and Health, Manchester Academic Health Science Centre, University of Manchester, Manchester, UK

**Keywords:** Parasitic infection, Interleukins, Infection, Parasite host response

Correction to: *Nature Communications* 10.1038/s41467-023-42918-8, published online 03 November 2023

The original version of this Article contained an error in Fig. 7, in which 3 numerical p values were missing from figure Panel E.

This has been corrected and the values included in Figure 7 panel E in both the PDF and HTML versions of the Article.

