# Supplementary material for: IL-17 signalling is critical for controlling subcutaneous adipose tissue dynamics and parasite burden during chronic murine Trypanosoma brucei infection
Source: Nat Commun. 2023 Nov 3;14:7070. doi: 10.1038/s41467-023-42918-8 (PMC10624677; doi:10.1038/s41467-023-42918-8)
Supplement: Supplementary file 1 — Supplementary Information [file 41467_2023_42918_MOESM1_ESM.pdf]

Supplementary Figures

Figure S1

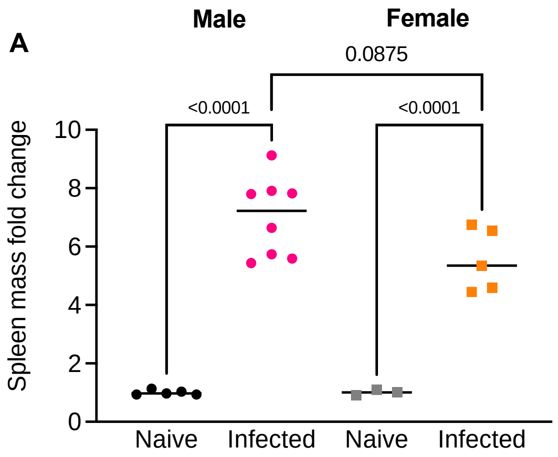

Supplementary Figure 1. *T. brucei* infection increases spleen mass in both male and female mice. Spleen weights were normalised to bodyweight and the fold change in weight calculated. Each point represents a biological replicate. Data were analysed using one-way ANOVA with Tukey post-hoc testing.

Figure S2

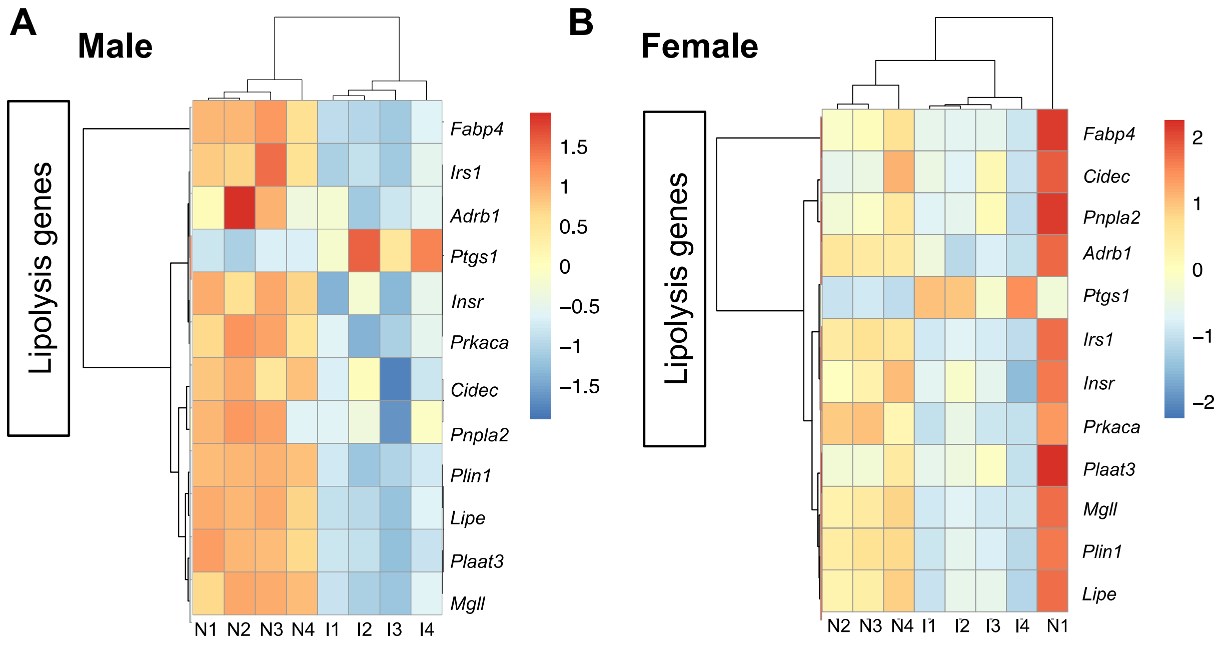

Supplementary Figure 2. *T. brucei* infection downregulates genes associated with lipolysis in male and female mice. Heatmaps, clustered by Euclidean distance, showing downregulation of lipolysis-related genes in male, scaled by Z-score (**A**) and female (**B**) mice during chronic *T. brucei* infection. Labels N1-N4 refer to naïve samples. Labels I1-I4 refer to infected samples.

Figure S3

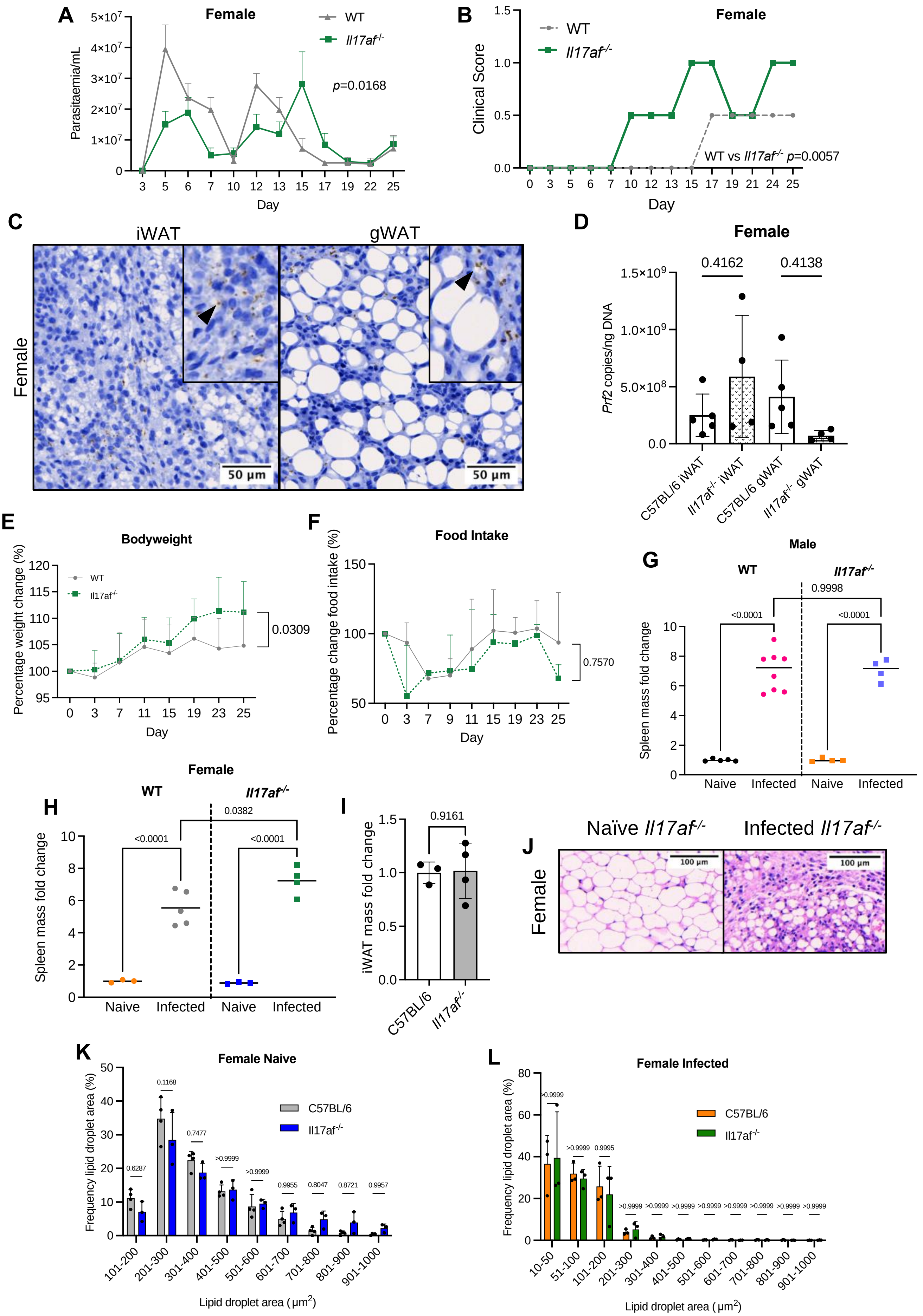

A
Female
WT
Il17af-/-
p=0.0168
Parasitaemia/mL
Day
B
Female
WT
Il17af-/-
Clinical Score
WT vs Il17af-/- p=0.0057
Day
C
iWAT
gWAT
Female
50 µm
50 µm
D
Female
0.4162
0.4138
Prf2 copies/ng DNA
C57BL/6 iWAT
Il17af-/- iWAT
C57BL/6 gWAT
Il17af-/- gWAT
E
Bodyweight
WT
Il17af-/-
0.0309
Percentage weight change (%)
Day
F
Food Intake
0.7570
Percentage change food intake (%)
Day
G
Male
WT
Il17af-/-
0.9998
<0.0001
<0.0001
Spleen mass fold change
Naive
Infected
Naive
Infected
H
Female
WT
Il17af-/-
0.0382
<0.0001
<0.0001
Spleen mass fold change
Naive
Infected
Naive
Infected
I
0.9161
iWAT mass fold change
C57BL/6
Il17af-/-
J
Naïve Il17af-/-
Infected Il17af-/-
Female
100 µm
100 µm
K
Female Naive
C57BL/6
Il17af-/-
Frequency lipid droplet area (%)
Lipid droplet area (µm2)
L
Female Infected
C57BL/6
Il17af-/-
Frequency lipid droplet area (%)
Lipid droplet area (µm2)

Supplementary Figure 3. **IL-17A/F does not drive weight loss in female mice.** (**A**) Number of parasites per mL of blood, measured using phase microscopy and the rapid "matching" method[77]. (**B**) Clinical scores of infected female mice. (**C**) Histological analysis of the iWAT and gWAT trypanosome colonisation, using HSP70 staining. (**D**) Parasite burden of iWAT and gWAT, which was measured by RT-qPCR of genomic DNA. (**E**) Percentage changes in body weight of female mice over the course of infection. (**F**) Percentage change in gross food intake. Each data point represents 2 cages (*n*=3-4 mice per cage). Spleen weights from males (**G**) and females (**H**) were normalised to bodyweight and the fold change in weight calculated. (**I**) iWAT mass at 25 days post-infection or in naïve mice. iWAT was dissected and weighed before normalising to body weight, to account for variation between biological replicates. (**J**) Representative histological H&E staining of iWAT showing adipocyte lipid droplets and immune infiltrate. (**K-L**) Analysis of lipid droplet area (μm^2) in naïve and infected females. *N*=6 biological replicates per group, from two independent experiments. Lipid droplets were measured from 3 distinct areas in each image and then combined for each biological replicate. Time series data were analysed using two-way repeated measures ANOVA with Sidak post-hoc testing. **D**, **G**, and **H** were analysed using a one-way ANOVA with Tukey's post-hoc testing. **I** was analysed using a student's t-test. Data for all panels are expressed as mean ±SD.

Figure S4

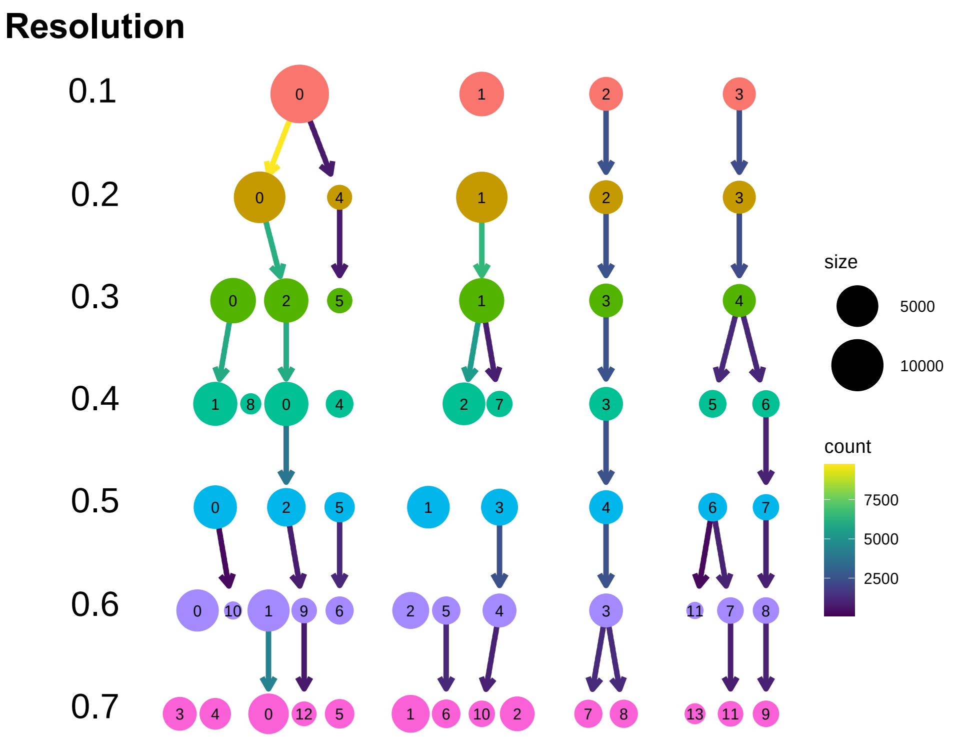

Supplementary Figure 4. Clustree analysis of single cell RNA sequencing data.

Figure S5

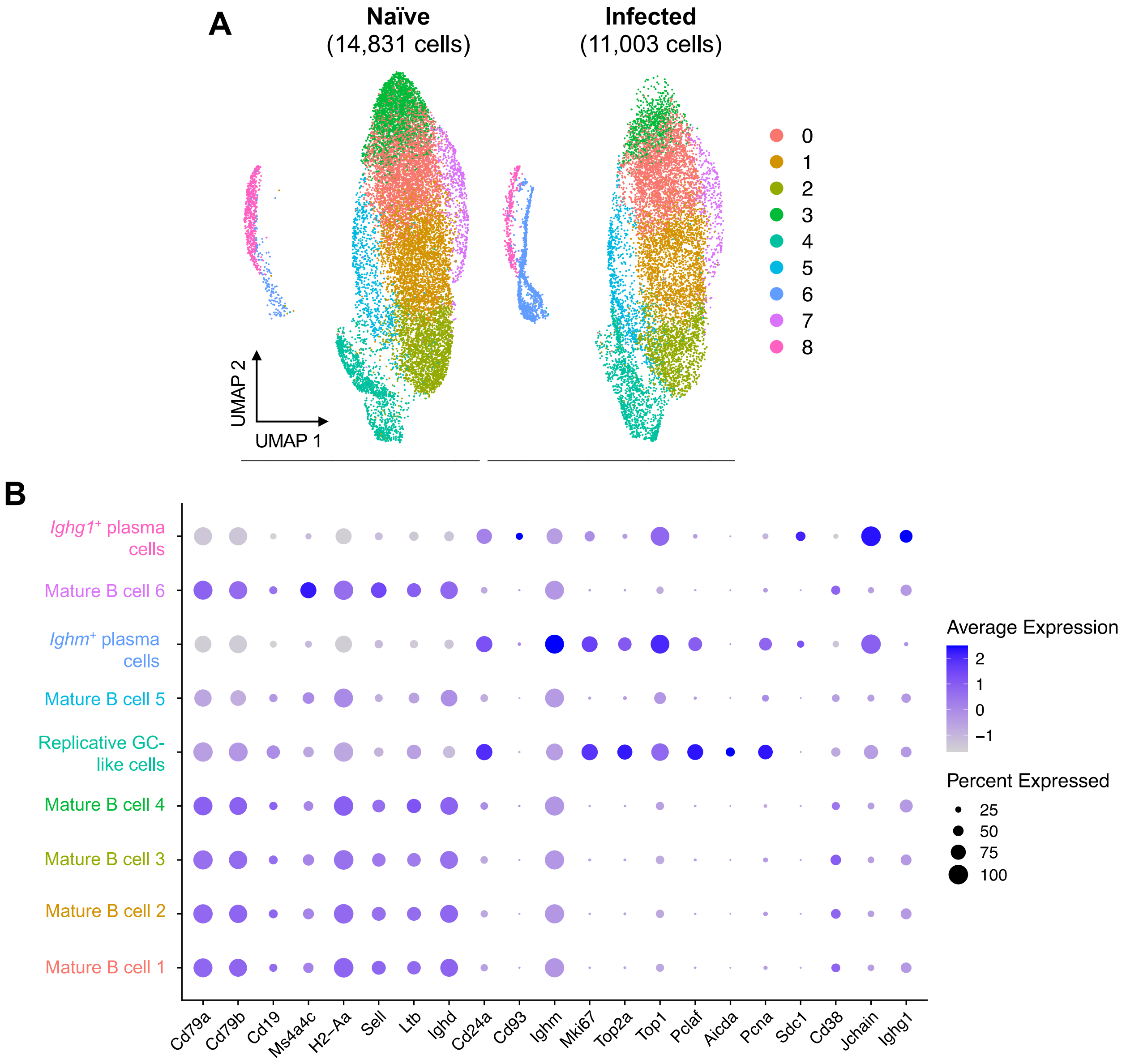

Supplementary Figure 5. Single cell analysis of B cell clusters from the iWAT of naïve and infected male mice. (**A**) UMAP of subclustered B cells. (**B**) Dot plot showing expression of genes used to identify specific populations of B cells.

Figure S6

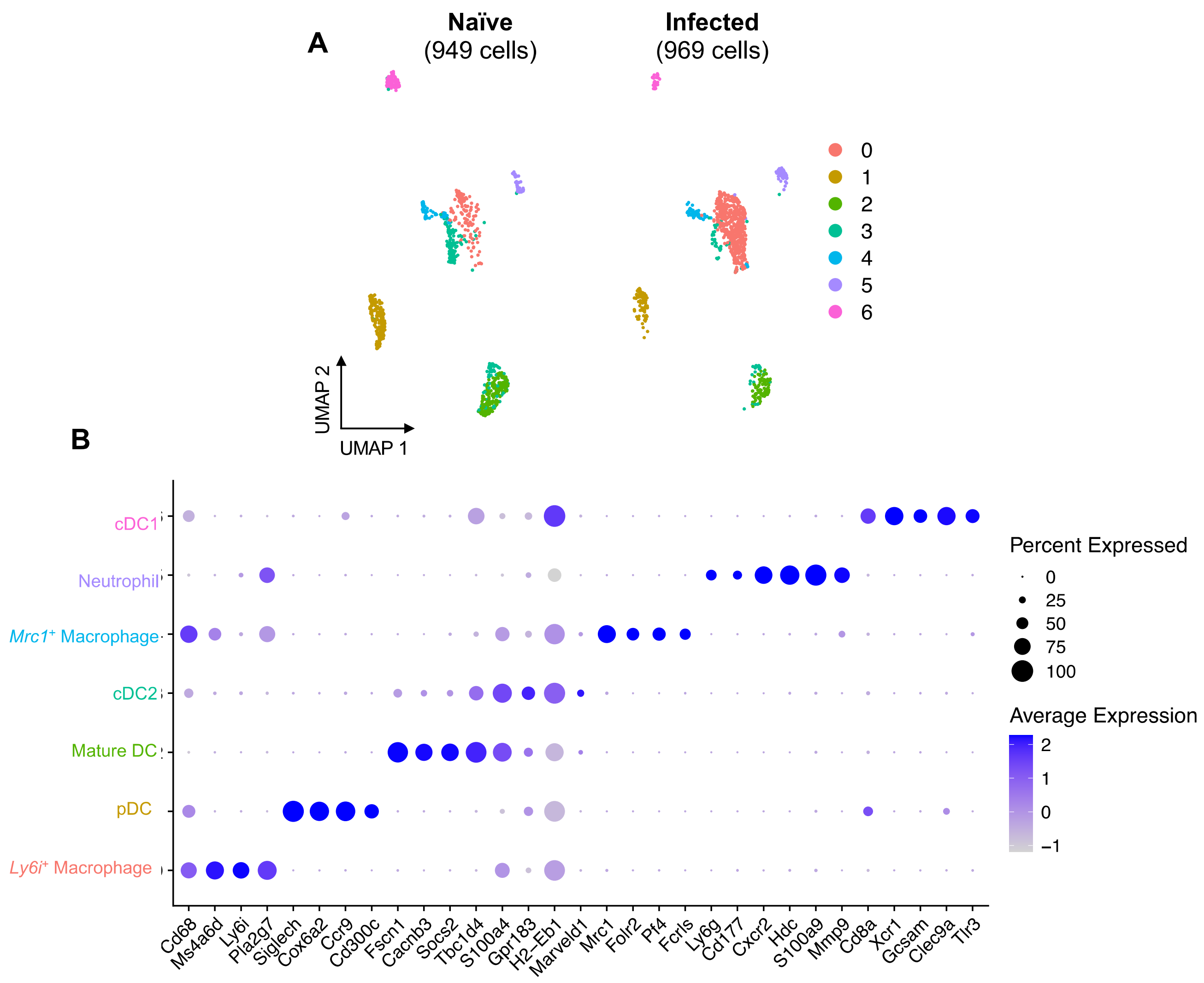

Supplementary Figure 6. Single cell analysis of myeloid cell clusters from the iWAT of naïve and infected male mice. (**A**) UMAP of subclustered myeloid cells. (**B**) Dot plot showing expression of genes used to identify specific populations of myeloid cells.

Figure S7

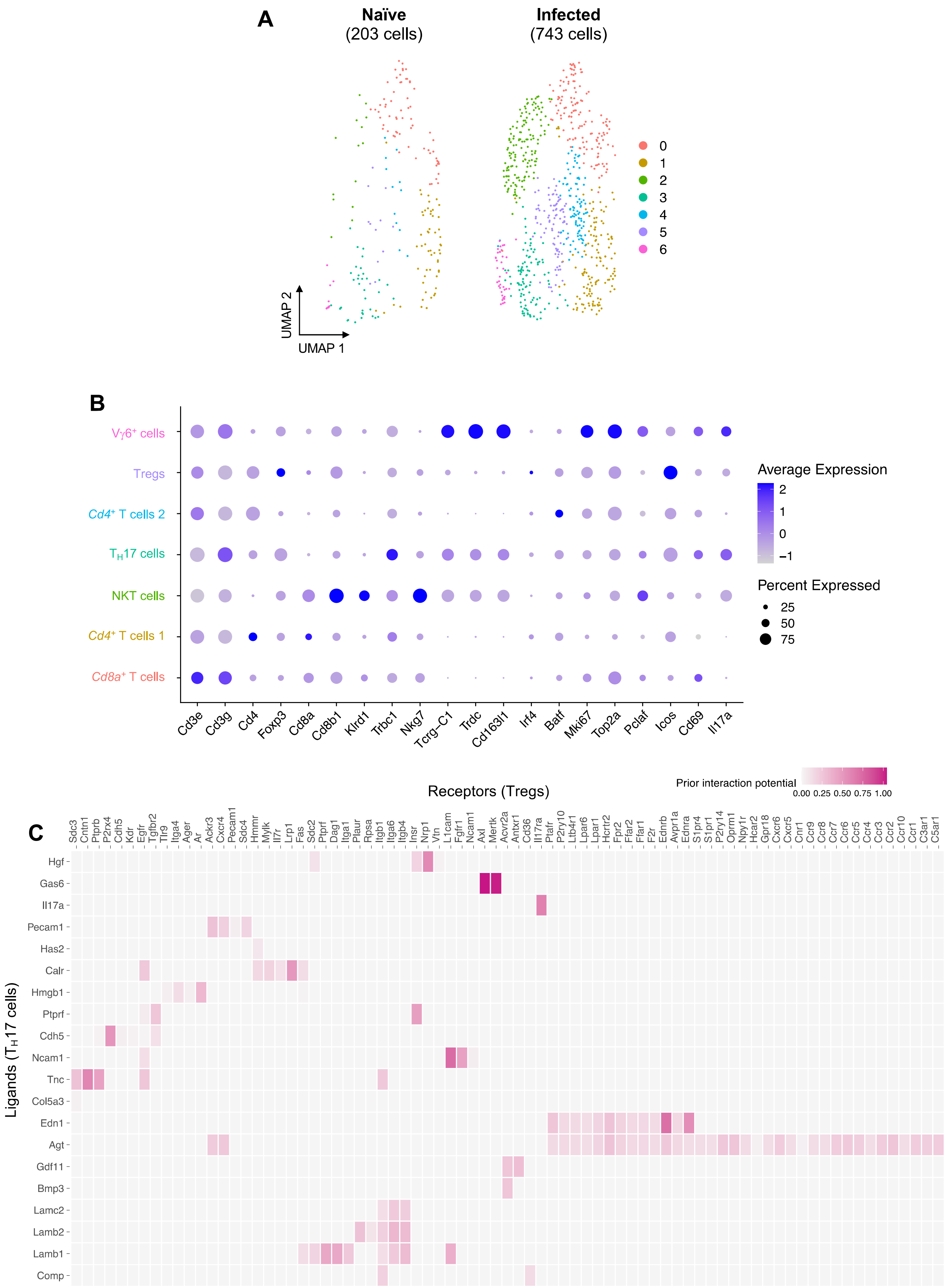
A
Naïve
(203 cells)
Infected
(743 cells)
UMAP 2
UMAP 1
0
1
2
3
4
5
6
B
Vγ6⁺ cells
Tregs
Cd4⁺ T cells 2
TH17 cells
NKT cells
Cd4⁺ T cells 1
Cd8a⁺ T cells
Cd3e Cd3g Cd4 Foxp3 Cd8a Cd8b1 Klrd1 Trbc1 Nkg7 Tcrg–C1 Trdc Cd163l1 Irf4 Batf Mki67 Top2a Pclaf Icos Cd69 Il17a
Average Expression
2
1
0
−1
Percent Expressed
25
50
75
C
Receptors (Tregs)
Prior interaction potential
0.00 0.25 0.50 0.75 1.00
Sdc3 Cntn1 Ptprb P2rx4 Cdh5 Kdr Egfr Tgfbr2 Tlr9 Itga4 Ager Ar Ackr3 Cxcr4 Pecam1 Sdc4 Hmmr Mylk Il7r Lrp1 Fas Sdc2 Ptprf Dag1 Itga1 Plaur Rpsa Itgb1 Itga6 Itgb4 Insr Nrp1 Vtn L1cam Fgfr1 Ncam1 Axl Mertk Acvr2a Antxr1 Cd36 Il17ra Ptafr P2ry10 Ltb4r1 Lpar6 Lpar1 Hcrtr2 Fpr2 Ffar2 Ffar1 F2r Ednrb Avpr1a Ednra S1pr4 S1pr1 P2ry14 Oprm1 Npy1r Hcar2 Gpr18 Cxcr6 Cxcr5 Cnr1 Ccr9 Ccr8 Ccr7 Ccr6 Ccr5 Ccr4 Ccr3 Ccr2 Ccr10 Ccr1 C3ar1 C5ar1
Ligands (TH17 cells)
Hgf
Gas6
Il17a
Pecam1
Has2
Calr
Hmgb1
Ptprf
Cdh5
Ncam1
Tnc
Col5a3
Edn1
Agt
Gdf11
Bmp3
Lamc2
Lamb2
Lamb1
Comp

Supplementary Figure 7. Single cell analysis of replicative T cell clusters from the iWAT of naïve and infected male mice. (**A**) UMAP of subclustered replicative T cells. (**B**) Dot plot showing expression of genes used to identify specific populations of replicative T cells. (**C**) Cell-cell communication analysis of T_H17 cells and Tregs.

Figure S8

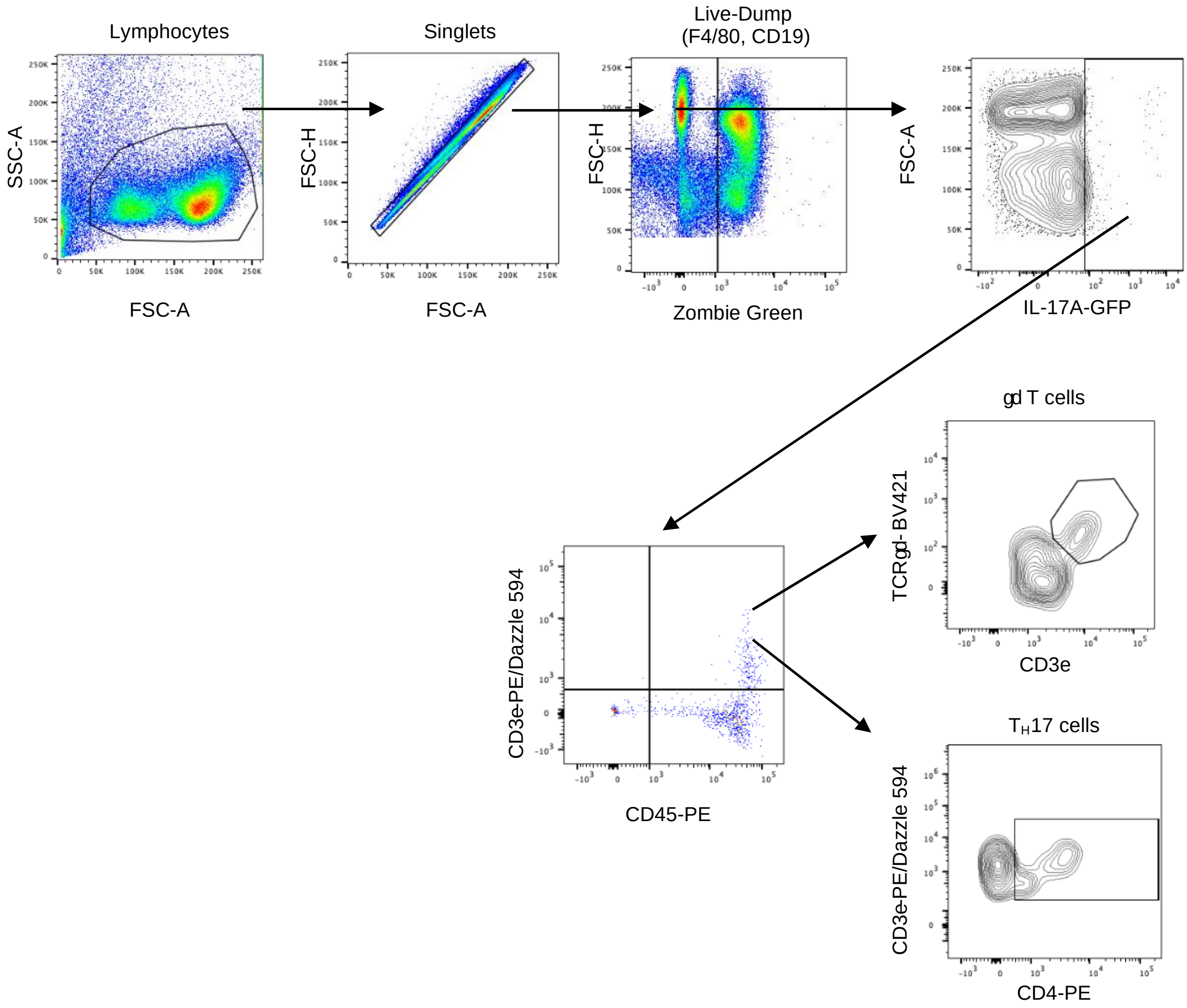

Supplementary Figure 8. Representative gating strategy for flow cytometry.

Figure S9

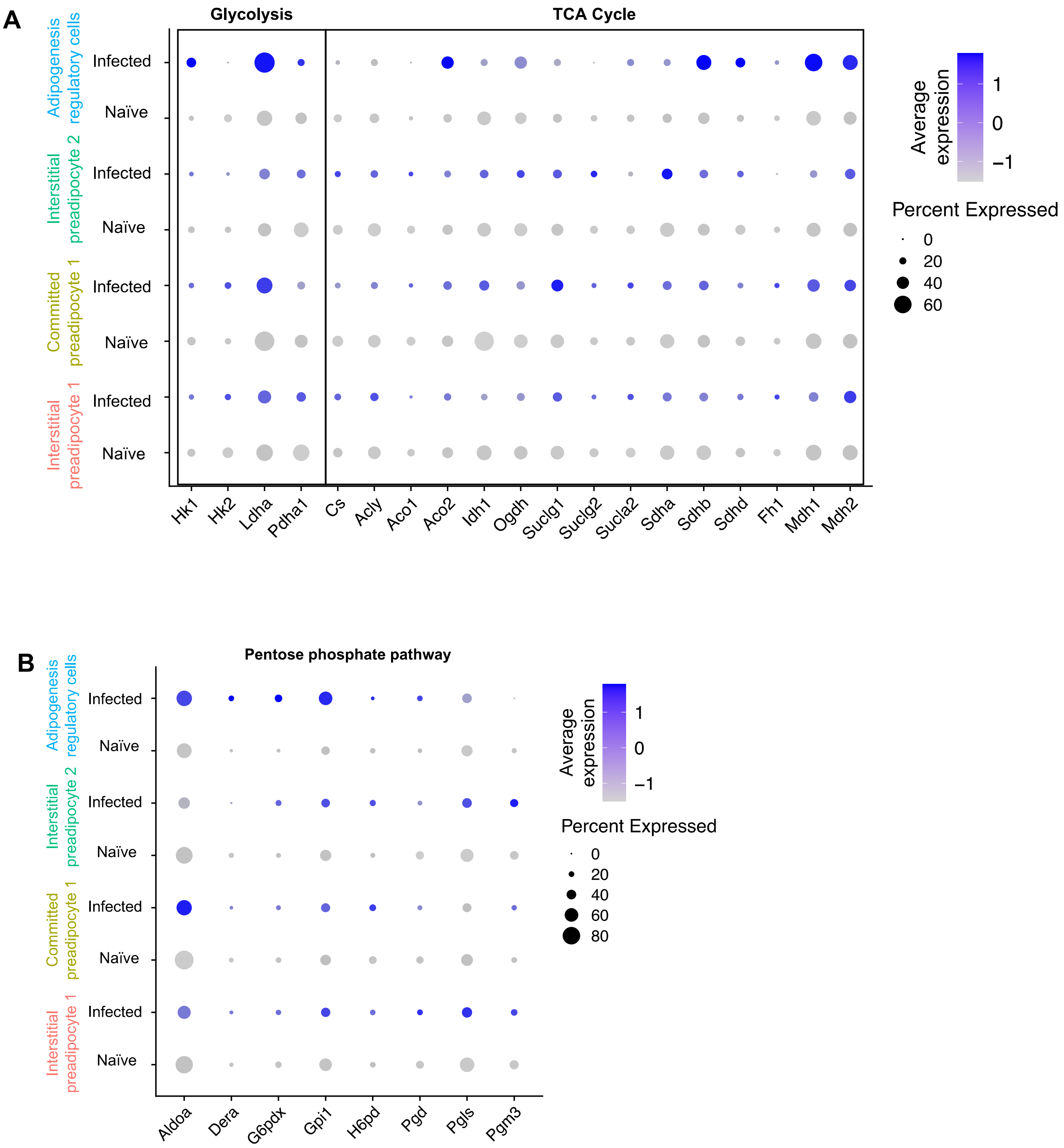

Supplementary Figure 9. Single cell analysis of genes associated with energy metabolism in preadipocytes during *T. brucei* infection. Dot plot showing expression of genes involved in glycolysis and the TCA cycle (**A**) or the pentose phosphate pathway (**B**). In the key, dots represent the percentage of cells that express a given marker, and the colour intensity represent the level of expression. Single cell data are comprised of one technical replicate per condition, each containing pooled cells from the iWAT of 5 male mice replicate.

Figure S10

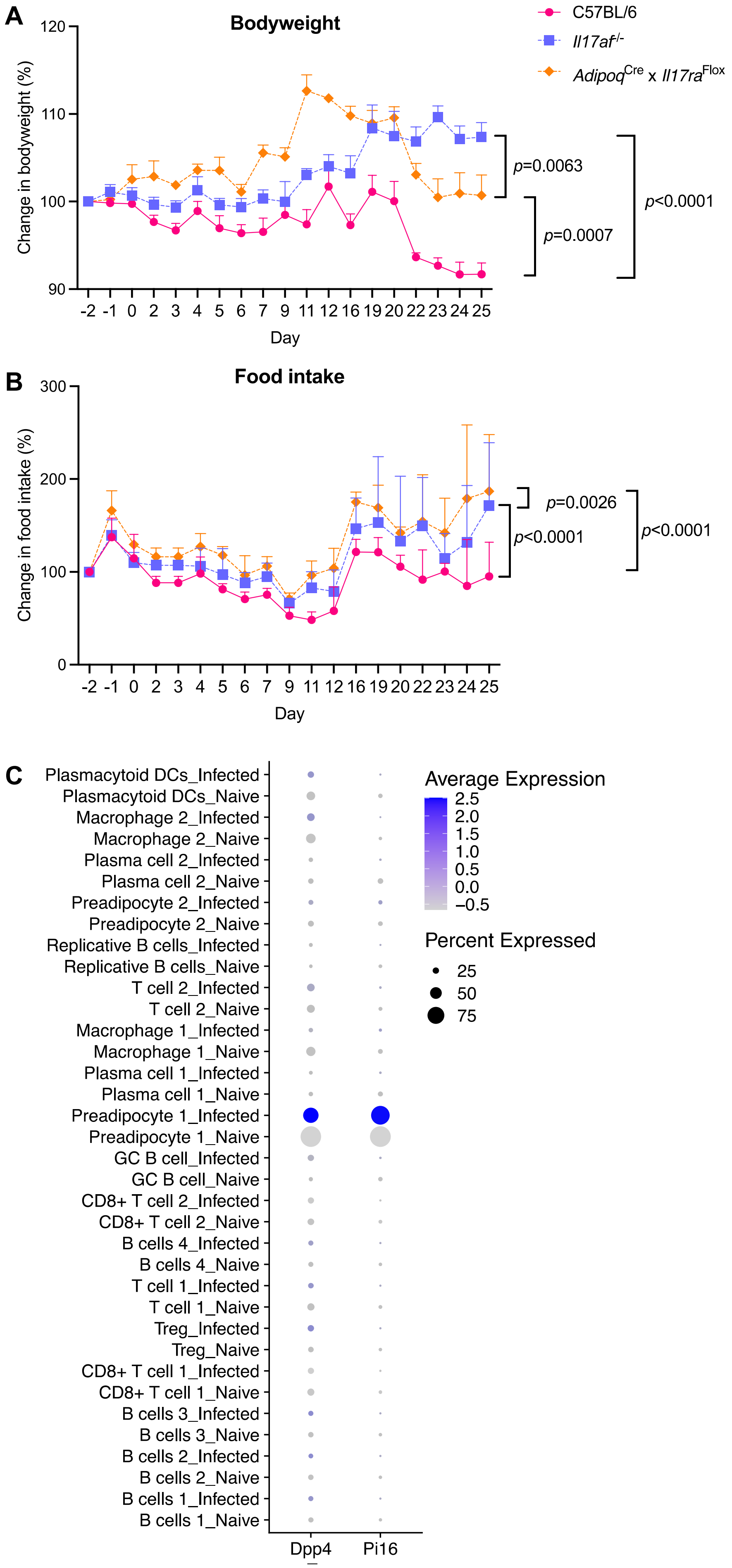
A
Bodyweight
C57BL/6
Il17af-/-
AdipoqCre x Il17raFlox
Change in bodyweight (%)
120
110
100
90
-2 -1 0 2 3 4 5 6 7 9 11 12 16 19 20 22 23 24 25
Day
p=0.0063
p=0.0007
p<0.0001
B
Food intake
Change in food intake (%)
300
200
100
0
-2 -1 0 2 3 4 5 6 7 9 11 12 16 19 20 22 23 24 25
Day
p=0.0026
p<0.0001
p<0.0001
C
Plasmacytoid DCs_Infected
Plasmacytoid DCs_Naive
Macrophage 2_Infected
Macrophage 2_Naive
Plasma cell 2_Infected
Plasma cell 2_Naive
Preadipocyte 2_Infected
Preadipocyte 2_Naive
Replicative B cells_Infected
Replicative B cells_Naive
T cell 2_Infected
T cell 2_Naive
Macrophage 1_Infected
Macrophage 1_Naive
Plasma cell 1_Infected
Plasma cell 1_Naive
Preadipocyte 1_Infected
Preadipocyte 1_Naive
GC B cell_Infected
GC B cell_Naive
CD8+ T cell 2_Infected
CD8+ T cell 2_Naive
B cells 4_Infected
B cells 4_Naive
T cell 1_Infected
T cell 1_Naive
Treg_Infected
Treg_Naive
CD8+ T cell 1_Infected
CD8+ T cell 1_Naive
B cells 3_Infected
B cells 3_Naive
B cells 2_Infected
B cells 2_Naive
B cells 1_Infected
B cells 1_Naive
Dpp4
Pi16
Average Expression
2.5
2.0
1.5
1.0
0.5
0.0
−0.5
Percent Expressed
25
50
75

Supplementary Figure 10. Comparison of weight loss and food intake in C57BL/6, *Il17af-/-* and *Adipoq*^{Cre} x *Il17ra*^{Flox} mice. (**A**) Three-way comparison of the bodyweight of single-housed infected C57BL/6, *Il17af*-/- and *Adipoq*^{Cre} x *Il17ra*^{Flox} mice. (**B**) Three-way comparison of the food intake of single-housed infected C57BL/6, *Il17af*-/- and *Adipoq*^{Cre} x *Il17ra*^{Flox} mice. (**C**) Dot plot from scRNAseq showing expression of *Dpp4* and *Pi16* across all cell populations. Time series data were analysed using two-way repeated measures ANOVA with Sidak post-hoc testing. Data for all panels are expressed as mean ±SD.
