# Supplementary material for: IL-17 signalling is critical for controlling subcutaneous adipose tissue dynamics and parasite burden during chronic murine Trypanosoma brucei infection
Source: Nat Commun. 2023 Nov 3;14:7070. doi: 10.1038/s41467-023-42918-8 (PMC10624677; doi:10.1038/s41467-023-42918-8)
Supplement: Supplementary file 3 — Description of Additional Supplementary Files [file 41467_2023_42918_MOESM3_ESM.pdf]

Description of Additional Supplementary Files

Supplementary Data 1. Metadata and serum glycerol and cytokine measurements from HAT patients. 1A) Metadata from healthy control and HAT patients in the Democratic Republic of Congo, and measurements of circulating glycerol. **1B**) Metadata from healthy control and HAT patients in Guinea, and measurements of circulating cytokines IL-17A, IFN-γ, and TNF-α.

Supplementary Data 2. Differential expression analyses of bulk RNA transcriptomic data from the inguinal white adipose tissue of mice infected with *Trypanosoma brucei*. 2A) Differential expression analysis of bulk transcriptomic data from the iWAT of male mice infected with *T. brucei* compared with naïve controls. **2B**) Differential expression analysis of bulk transcriptomic data from the iWAT of female mice infected with *T. brucei* compared with naïve controls.

Supplementary Data 3. Pathway enrichment analyses of bulk RNA transcriptomic datasets from the inguinal white adipose tissue of mice infected with *Trypanosoma brucei*. 3A) KEGG enrichment analysis of upregulated genes from the iWAT of male mice infected with *T. brucei* compared with naïve controls. **3B**) KEGG enrichment analysis of downregulated genes from the iWAT of male mice infected with *T. brucei* compared with naïve controls. **3C**) KEGG enrichment analysis of upregulated genes from the iWAT of female mice infected with *T. brucei* compared with naïve controls. **3D**) KEGG enrichment analysis of downregulated genes from the iWAT of female mice infected with *T. brucei* compared with naïve controls. Pathways were considered significantly enriched where the false discovery rate (FDR) was $p<0.05$.

Supplementary Data 4. Expression of genes related to T helper 17 cell expression in the inguinal white adipose tissue of mice infected with *Trypanosoma brucei*. 4A) The log2fold change in gene expression in male and female mice. Genes were selected from those listed in the pathways described in Supplementary Data 3. Log2fold change and adjusted *p* value were selected from Supplementary Data 2. Changes in expression were considered significant with an adjusted *p* value of <0.05.

Supplementary Data 5. Overview of the mouse inguinal white adipose tissue single cell transcriptomics during chronic *Trypanosoma brucei* infection. 5A) Quality control including mean reads per cell and median genes per cell before and after filtering out low quality cell types.

Supplementary Data 6. Description of cell counts in the clusters generated from the single cell transcriptomic analysis of the inguinal white adipose tissue during *Trypanosoma brucei* infection. 6A) Overview of the major cell types detected in the single cell dataset and the numbers of each of these cells in infected of naïve samples.

Supplementary Data 7. Differential expression analyses of scRNAseq clusters from inguinal white adipose tissue during *Trypanosoma brucei* infection. 7A) Differential expression analyses for each cell cluster are presented as enriched in the infected versus the naïve sample. Expression was classified as significant where the adjusted *p* value (p_val_adj) was <0.05.
