# Supplementary material for: IL-17 signalling is critical for controlling subcutaneous adipose tissue dynamics and parasite burden during chronic murine Trypanosoma brucei infection
Source: Nat Commun. 2023 Nov 3;14:7070. doi: 10.1038/s41467-023-42918-8 (PMC10624677; doi:10.1038/s41467-023-42918-8)
Supplement: Supplementary file 11 — Reporting Summary [file 41467_2023_42918_MOESM11_ESM.pdf]

nature portfolio

Corresponding author(s): Matthew C. Sinton, Juan F. Quintana

Last updated by author(s): 9th October 2023

Reporting Summary

Nature Portfolio wishes to improve the reproducibility of the work that we publish. This form provides structure for consistency and transparency in reporting. For further information on Nature Portfolio policies, see our Editorial Policies and the Editorial Policy Checklist.

Statistics

For all statistical analyses, confirm that the following items are present in the figure legend, table legend, main text, or Methods section.

n/a	Confirmed	
☐	☒	The exact sample size (*n*) for each experimental group/condition, given as a discrete number and unit of measurement
☐	☒	A statement on whether measurements were taken from distinct samples or whether the same sample was measured repeatedly
☐	☒	The statistical test(s) used AND whether they are one- or two-sided<br>*Only common tests should be described solely by name; describe more complex techniques in the Methods section.*
☒	☐	A description of all covariates tested
☐	☒	A description of any assumptions or corrections, such as tests of normality and adjustment for multiple comparisons
☐	☒	A full description of the statistical parameters including central tendency (e.g. means) or other basic estimates (e.g. regression coefficient) AND variation (e.g. standard deviation) or associated estimates of uncertainty (e.g. confidence intervals)
☐	☒	For null hypothesis testing, the test statistic (e.g. *F*, *t*, *r*) with confidence intervals, effect sizes, degrees of freedom and *P* value noted<br>*Give P values as exact values whenever suitable.*
☒	☐	For Bayesian analysis, information on the choice of priors and Markov chain Monte Carlo settings
☒	☐	For hierarchical and complex designs, identification of the appropriate level for tests and full reporting of outcomes
☒	☐	Estimates of effect sizes (e.g. Cohen's *d*, Pearson's *r*), indicating how they were calculated

Our web collection on statistics for biologists contains articles on many of the points above.

Software and code

Policy information about availability of computer code

Data collection	Single cell RNA sequencing was conducted on an Illumina Novaseq 6000 sequencer by Glasgow Polyomics. Fastq sequence files were de-multiplexed, aligned, and annotated using a reference combined mouse genome (mmu10;https://ncbi.nlm.nih.gov/assembly/GCF_000001635.20/) and Cell Ranger software (vCR6.1; single cell RNA sequencing). Gene expression was counted using unique molecular identifier barcodes, and gene-cell matrices were constructed. Bulk RNA sequencing was conducted on an Illumina Novaseq 6000 sequencer by Novogene (Cambridge, UK). Raw reads were processed by Novogene using proprietary scripts in Perl. An index of the reference genome (Genome Reference Consortium Mouse Build; GRCm39) was constructed, and reads were aligned, both using Hisat2 (v2.0.5). Read numbers were counted using featureCounts (v. 1.5.0-p3). FACSDIVA software (v9.0) was used for acquisition of flow cytometry data. Mass cytometry samples were acquired using a CyTOF XT (Standard BioTools)
Data analysis	The following packages were used to analyse the single cell data: R (v4.2.1), Seurat (v4.1.0), sctransform (v0.3.3), RcolorBrewer (v1.1.2). For bulk RNA sequencing, differential expression analyses conducted using DESeq2 (v. 1.20.0). FlowJo (v10.8.2) was used to analyse flow cytometry data. Mass cytometry data were analysed using the Cytobank platform (Beckman Coulter Life Sciences). The scripts used to generate single cell data in this study and the processed data are available at Zenodo (https://zenodo.org/record/7966849).

For manuscripts utilizing custom algorithms or software that are central to the research but not yet described in published literature, software must be made available to editors and reviewers. We strongly encourage code deposition in a community repository (e.g. GitHub). See the Nature Portfolio guidelines for submitting code & software for further information.

Data

Policy information about availability of data

All manuscripts must include a data availability statement. This statement should provide the following information, where applicable:

- Accession codes, unique identifiers, or web links for publicly available datasets
- A description of any restrictions on data availability
- For clinical datasets or third party data, please ensure that the statement adheres to our policy

The GEO accession number for raw bulk transcriptomic sequencing and processed data reported in this paper is GSE210600 (https://www.ncbi.nlm.nih.gov/geo/query/acc.cgi?acc=GSE210600). The GEO accession number for the raw scRNAseq and processed data reported in this paper is GSE233312 (https://www.ncbi.nlm.nih.gov/geo/query/acc.cgi?acc=GSE233312). Source data are provided with this paper.

Research involving human participants, their data, or biological material

Policy information about studies with human participants or human data. See also policy information about sex, gender (identity/presentation), and sexual orientation and race, ethnicity and racism.

Reporting on sex and gender	For human samples collected from Guinea: the sex ratio (male:female) of the study population was 1.41 (124:88) and the mean age (range) was 32.76 (5-85 years). We received a subset of these samples and due to the limited number we did not stratify by sex.<br><br>For human samples collected from the Democratic Republic of Congo: of the 233 samples collected, there was a sex ratio (male:female) of 0.63. We received a subset of these samples and due to the limited number we did not stratify by sex.
Reporting on race, ethnicity, or other socially relevant groupings	Samples were collected from either Guinea or the Democratic Republic of Congo, where HAT is endemic.
Population characteristics	For human samples from Guinea: samples were collected from the TrypanoGEN Biobank. In the initial study where samples were collected, serum was collected from 212 patients. The cohort consisted of healthy endemic controls n=46 (21.70%) and T.b. gambiense-infected individuals that were subdivided into two phenotypes: 1) patients with active HAT n=141 (66.51%) of whom 33 (23.40%) were sampled again after treatment, and 2) individuals with latent infections n=25 (11.79%) who tested positive in serology, but were negative upon microscopic examination and exhibited few or no symptoms. These individuals were followed up for a period of at least two years and none developed detectable blood parasitaemia during their follow up despite remaining positive in serology. The sex ratio (male:female) of the study population was 1.41 (124:88) and the mean age range was 32.76 (5-85 years). The samples that were used in this study were from patients >18 years of age, and either healthy endemic controls or patients with active HAT.<br><br>For human samples collected from the Democratic Republic of Congo: Subjects were aged at least 12 years old and were enrolled in this study from July 2013 to March 2016 during the active screening campaigns of the mobile team of the National Programme of Control of Human African Trypanosomiasis. Individuals who could not provide a sufficient volume of blood (less than 9 mL) or were under 12 years old were excluded. Previously treated patients were not included as controls. Of the 233 samples collected there was a sex ratio (male:female) of 0.63.
Recruitment	For human samples from Guinea: Participants were identified during surveys organised by the Guinean National Control Programme (NCP) between 2007 and 2011 from three HAT foci in Guinea (Dubreka, Boffa and Forecariah) according to the WHO and NCP policies. All participants were informed of the objective of the study in their own language and signed an informed consent form.<br><br>For human samples from the Democratic Republic of Congo: The study was undertaken in two stages: 1) a screen of 96 candidate loci in 233 subjects and 2) a validation study of two loci that were suggestively positive in the first round in 594 additional subjects. Subjects were aged at least 12 years old and were enrolled in this study from July 2013 to March 2016 during the active screening campaigns of the mobile team of the National Program of Control of Human African Trypanosomiasis (PNLTHA). Participants were recruited in their respective villages during active screening campaigns.
Ethics oversight	Comité Consultative de Déontologie et d'Ethique (CCDE) of the Institut de Recherche pour le Développement (approval number 1—22/04/2013); Democratic Republic of Congo National Ministry of Public Health (approval number 1/2013); University of Glasgow (approval number: 200120043)

Note that full information on the approval of the study protocol must also be provided in the manuscript.

Field-specific reporting

Please select the one below that is the best fit for your research. If you are not sure, read the appropriate sections before making your selection.

[x] Life sciences [ ] Behavioural & social sciences [ ] Ecological, evolutionary & environmental sciences

For a reference copy of the document with all sections, see nature.com/documents/nr-reporting-summary-flat.pdf

Life sciences study design

All studies must disclose on these points even when the disclosure is negative.

Sample size	The sample size for bulk transcriptomics experiments presented in this study was 4 biological replicates per group. For scRNAseq, 1 technical replicate was used per group, and each technical replicate was generated from 5 mice. For in vivo experiments, we used a sample size of at least 3 animals per experimental condition. For all experiments in this study, sample size was based on measures of variance from historical data in the laboratory, and is enough to detect differences between experimental and control groups at a 5% significance level and 90% power.
Data exclusions	No data were excluded from the analysis.
Replication	All experiments were conducted using independent biological replicates and were repeated 1-3 times. Where experiments were repeated, replication was successful. The data obtained from the single cell experiments (n=1 biological replicate/group, performed once) were succesfully replicated in single independent in vivo experiments (e.g., expansion of Vg6 T cells in the iWAT).
Randomization	Mice were randomly assigned to cages for all experiments by technical staff, who were only aware of the sex or genotype required for the experiments performed, Cages were then selected at random for infections or to act as controls.<br><br>Randomisation of scRNAseq/bulk RNAseq was not appropriate as both naive and infected animals were processed in parallel and required additional labelling for downstream analysis (e.g., flow cytometry analysis, H&E analysis). Randomisation of in vivo experiments was not appropriate as the infected animals required to be closely monitored for the development of clinical adverse effects. Animals used for flow cytometry analysis described here as validation for single cell were randomly allocated into cages.
Blinding	Blinding of scRNA was not appropriate as data processing and analysis need to be carried out appropriately and differentially for biological between experimental conditions. Measurements associated with bodyweight, food intake parasitaemia and clinical scoring were conducted by a trained scientist blinded to the study. For flow cytometry analysis, blinding was not carried out in order to allow inclusion of appropriate measurements of background intensity and signal adjustments during acquisition. For histological studies, measurements of parasite burden, and qPCR, the scientists performing the analyses were blinded to the conditions that they were analysing.

Reporting for specific materials, systems and methods

We require information from authors about some types of materials, experimental systems and methods used in many studies. Here, indicate whether each material, system or method listed is relevant to your study. If you are not sure if a list item applies to your research, read the appropriate section before selecting a response.

Materials & experimental systems

n/a	Involved in the study	
☐	☒	Antibodies
☒	☐	Eukaryotic cell lines
☒	☐	Palaeontology and archaeology
☐	☒	Animals and other organisms
☒	☐	Clinical data
☒	☐	Dual use research of concern
☒	☐	Plants

Methods

n/a	Involved in the study	
☒	☐	ChIP-seq
☐	☒	Flow cytometry
☒	☐	MRI-based neuroimaging

Antibodies

Antibodies used	For mass cytometry: Maxpar® Mouse Sp/LN Phenotyping Panel Kit (Standard BioTools, 201306), containing the following antibodies: Ly6G/C [Gr1] (141Pr, clone RB6-8C5, 1/100), CD11c (142Nd, clone N418, 1/100), CD69 (145Nd, clone H1.2F3, 1/100), CD45 (147Sm, clone 30-F11, 1/200), CD11b (148Nd, clone M1/70, 1/100), CD19 (149Sm, clone 6D5, 1/100), CD3 (152Sm, clone 145-2C11, 1/100), TCR (169Tm, clone H57-597, 1/100), CD44 (171Yb, clone IM7, 1/100), CD4 (172Yb, clone RM4-5, 1/100). Additionally, we added the following antibodies (Standard BioTools) to our panel: TCRgd (159Tb, clone GL3, 1/100, 3159012C), and CD27 (150Nd, clone LG.3A10, 1/100, 3150017B). Cell pellets were resuspended in intracellular antibody cocktail (Standard BioTools): IL-17A (174Yb, clone TC11-18H10.1, 1/100, 3174002C) and IFN (165Ho, clone XMG1.2, 1/100, 3165003C).<br><br>For flow cytometry: Viability staining was performed using Zombie Green Fixable Viability Dye at 1/1000 dilution (BioLegend), and then samples were stained at 1/400 dilution with the following antibodies from BioLegend: F4/80-PE/Cy7 (clone BM8, 1/400), CD19-PE/Cy7 (clone 6D5, 1/400), TCR-BV421 (clone GL3, 1/400), CD27-APC (clone LG.3A10, 1/400), CD45-PE or CD45-BV421 (clone 30-F11, 1/400), CD3-PE/Dazzle594 (clone 145-2C11, 1/400) or CD4-PE (clone RM4-4, 1/400).<br><br>The HSP70 antibody used for histological staining was a kind gift from Professor James D. Bangs.
Validation	All antibodies for flow cytometry were used at the recommended concentration by the manufacturer and tested in pilot titration experiments. The antibodies used for imaging were used exactly as recommended by the manufacturer, including the concentration.

Validation statements can be found on the manufacturer's websites:

https://store.standardbio.com/Cytometry/ConsumablesandReagentsCytometry/MaxparPanelKits/Maxpar%C2%AE%20Mouse%20Sp-LN%20Phenotyping%20Panel%20Kit-%2016%20Marker%E2%80%9425%20Tests
https://store.standardbio.com/Cytometry/ConsumablesandReagentsCytometry/MaxparAntibodies/Anti-Mouse%20TCRgd%20-GL3-159Tb%E2%80%94100%20Tests
https://store.standardbio.com/ccrz__ProductDetails?refURL=http%3A%2F%2Fstore.fluidigm.com%2FCytometry%2FConsumablesandReagentsCytometry%2FMaxparAntibodies%2FAnti-Human-Mouse%2520CD27%2520-LG-3A10-150Nd%25E2%2580%2594100%2520Tests&refURL=http%3A%2F%2Fstore.fluidigm.com%2FCytometry%2FConsumablesandReagentsCytometry%2FMaxparAntibodies%2FAnti-Human-Mouse%2520CD27%2520-LG-3A10-150Nd%25E2%2580%2594100%2520Tests&seoid=Anti-Human-Mouse+CD27+-LG-3A10-150Nd%E2%80%94100+Tests&sku=3150017B
https://store.standardbio.com/Cytometry/ConsumablesandReagentsCytometry/MaxparAntibodies/Anti-Mouse%20IL-17A%20-TC11-18H10-1-169Tm%E2%80%94100%20Tests
https://store.standardbio.com/Cytometry/ConsumablesandReagentsCytometry/MaxparAntibodies/Anti-Mouse%20IFNg%20-XMG1-2-165Ho%E2%80%94100%20Tests
https://www.biolegend.com/en-us/search-results/zombie-green-fixable-viability-kit-9340?Clone=&gclid=CjwKCAjwyY6pBhA9EiwAMzmfwSLjRNUOONqXy-u-kS4u9PLz8N9lRdIGdmhOeWLUGqHFZu_nPe7PYxoCLdMQAvD_BwE
https://www.biolegend.com/en-gb/products/pe-cyanine7-anti-mouse-f4-80-antibody-4070
https://www.biolegend.com/en-gb/products/pe-cyanine7-anti-mouse-cd19-antibody-1907?GroupID=BLG10556
https://www.biolegend.com/de-at/products/brilliant-violet-421-anti-mouse-tcr-gamma-delta-antibody-7249?GroupID=BLG3687
https://www.biolegend.com/fr-ch/products/apc-anti-mouse-rat-human-cd27-antibody-4395?GroupID=BLG10664
https://www.biolegend.com/fr-lu/products/brilliant-violet-421-anti-mouse-cd45-antibody-7253
https://www.biolegend.com/fr-fr/products/pe-dazzle-594-anti-mouse-cd3epsilon-antibody-10066?Clone=145-2C11
https://www.biolegend.com/en-gb/products/pe-anti-mouse-cd4-antibody-2499?GroupID=BLG4745

Animals and other research organisms

Policy information about studies involving animals; ARRIVE guidelines recommended for reporting animal research, and Sex and Gender in Research

Laboratory animals	Eight- to ten-week-old Adipoq-cre (JAX, stock 028020) or Il17ratm2.1Koll/J (JAX, stock 031000) were purchased from The Jackson Laboratory. These mice were crossed to generate AdipoqCre x Il17raFlox mice, with an adipocyte specific deletion of the IL-17A receptor. Six to eight weeks old male or female C57BL/6J (JAX, stock 000664), Il17af-/- (JAX, stock 034140), or IL-17A GFP reporter mice (JAX, stock 018472) were also purchased. At >10 weeks old, mice were randomly allocated to control or treatment groups by animal unit technical staff. Animals were housed on a 12 h light-dark cycle and fed ad libitum. Room temperature was between 20-24C and humidity was between 50-70%.
Wild animals	No wild animals were used in this study
Reporting on sex	Male and female mice were used in this study, and this was documented throughout this study.
Field-collected samples	No field samples were used in this study.
Ethics oversight	All animal experiments were approved by the University of Glasgow Ethical Review Committee and performed in accordance with the home office guidelines, UK Animals (Scientific Procedures) Act, 1986 and EU directive 2010/63/EU. All experiments were conducted under SAPO regulations and UK Home Office project licence number PC8C3B25C and PP4863348 to Dr Jean Rodgers and Professor Annette MacLeod, respectively.

Note that full information on the approval of the study protocol must also be provided in the manuscript.

Plants

Seed stocks	*Report on the source of all seed stocks or other plant material used. If applicable, state the seed stock centre and catalogue number. If plant specimens were collected from the field, describe the collection location, date and sampling procedures.*
Novel plant genotypes	*Describe the methods by which all novel plant genotypes were produced. This includes those generated by transgenic approaches, gene editing, chemical/radiation-based mutagenesis and hybridization. For transgenic lines, describe the transformation method, the number of independent lines analyzed and the generation upon which experiments were performed. For gene-edited lines, describe the editor used, the endogenous sequence targeted for editing, the targeting guide RNA sequence (if applicable) and how the editor was applied.*
Authentication	*Describe any authentication procedures for each seed stock used or novel genotype generated. Describe any experiments used to assess the effect of a mutation and, where applicable, how potential secondary effects (e.g. second site T-DNA insertions, mosiacism, off-target gene editing) were examined.*

Flow Cytometry

Plots

Confirm that:

- [x] The axis labels state the marker and fluorochrome used (e.g. CD4-FITC).
- [x] The axis scales are clearly visible. Include numbers along axes only for bottom left plot of group (a 'group' is an analysis of identical markers).
- [x] All plots are contour plots with outliers or pseudocolor plots.
- [x] A numerical value for number of cells or percentage (with statistics) is provided.

Methodology

Sample preparation	Infected animals and naive controls were anesthetized with isoflurane and perfused transcardially with 25-30 ml of ice-cold 1X PBS containing 0.025% (wt/vol) EDTA. iWAT pads were excised and the inguinal lymph node was removed. The iWAT was dissociated using an Adipose Tissue Dissociation Kit, mouse and rat (Miltenyi Biotec) with a gentleMACS’" Octo Dissociator with Heaters (Miltenyi Biotec) as per the manufacturer‘s recommendations. Digested tissue was then passed through a 70 km and then a 40 km nylon mesh filter, which were washed with DMEM. The suspension was then centrifuged at 400 x g at 4°C for 5 minutes to isolate the immune cell fraction and remove adipocytes. The resulting suspension was seeded on a 96-well plate and stimulated with 1X Cell Activation Cocktail containing phorbol 12-myristate 13-acetate (PMA), lonomycin, and Brefeldin A (BioLegend) for 3h at 37 C and 5% CO_2. For flow cytometry analysis, single cell suspensions were resuspended in ice-cold FACS buffer (2 mM EDTA, 5 U/ml DNAse I, 25 mM HEPES and 2.5% foetal calf serum (FCS) in 1X PBS) and stained for extracellular markers at 1:400 dilution. Samples were run on a flow cytometer LSRFortessa (BD Biosciences) and analysed using FlowJo software version 10 (Treestar).
Instrument	BD Fortessa Flow Cytometre (BD Biosciences)
Software	FACS Diva software (v9.0) and FlowJo (vl0.8.2) (BD).
Cell population abundance	25 µL of liquid compensation beads (BD Biosciences, 335925) were added to allow for quantification of absolute cell numbers.
Gating strategy	Unstained controls and isotype controls were used to identify background staining levels and determine gate placement. Doublets were excluded based on linearity of FSC-A and FSC-H. From singlets, live cells were identified as the fixable Zombie dye. T cell lymphocytes were detected by co-expression of CD45 and CD3e, and further subset into either gdT cells (TCRgd+), or conventional T cells (TCRgd-). The conventional population was further divided into either CD4 or CD8 T cells.

- [x] Tick this box to confirm that a figure exemplifying the gating strategy is provided in the Supplementary Information.
